# Implications of Change/Stability Patterns in Children’s Non-symbolic and Symbolic Magnitude Judgment Abilities Over One Year: A Latent Transition Analysis

**DOI:** 10.3389/fpsyg.2019.00441

**Published:** 2019-03-05

**Authors:** Cindy S. Chew, Jason D. Forte, Robert A. Reeve

**Affiliations:** Melbourne School of Psychological Sciences, University of Melbourne, Parkville, VIC, Australia

**Keywords:** non-symbolic and symbolic magnitude ability profiles, stability and change patterns, longitudinal analysis, visuospatial working memory, naming number ability, latent transition analysis

## Abstract

Non-symbolic magnitude abilities are often claimed to support the acquisition of symbolic magnitude abilities, which, in turn, are claimed to support emerging math abilities. However, not all studies find links between non-symbolic and symbolic magnitude abilities, or between them and math ability. To investigate possible reasons for these different findings, recent research has analyzed differences in non-symbolic/symbolic magnitude abilities using latent class modeling and has identified four different magnitude ability profiles residing within the general magnitude ability distribution that were differentially related to cognitive and math abilities. These findings may help explain the different patterns of findings observed in previous research. To further investigate this possibility, we (1) attempted to replicate earlier findings, (2) determine whether magnitude ability profiles remained stable or changed over 1 year; and (3) assessed the degree to which stability/change in profiles were related to cognitive and math abilities. We used latent transition analysis to investigate stability/changes in non-symbolic and symbolic magnitude abilities of 109 5- to 6-year olds twice in 1 year. At Time 1 and 2, non-symbolic and symbolic magnitude abilities, number transcoding and single-digit addition abilities were assessed. Visuospatial working memory (VSWM), naming numbers, non-verbal IQ, basic RT was also assessed at Time 1. Analysis showed stability in one profile and changes in the three others over 1 year. VSWM and naming numbers predicted profile membership at Time 1 and 2, and profile membership predicted math abilities at both time points. The findings confirm the existence of four different non-symbolic–symbolic magnitude ability profiles; we suggest the changes over time in them potentially reflect deficit, delay, and normal math developmental pathways.

## Introduction

Magnitude representation ability is as an important component of children’s math ability (Siegler, 2016). Near-identical error and RT response signatures for non-symbolic magnitude judgments and symbolic magnitude judgments is claimed to reflect a common underlying representation – the approximate number system (ANS) where magnitudes are ordered akin to a mental number line (Moyer and Landauer, 1967; Feigenson et al., 2004; Gebuis et al., 2009; Izard et al., 2009; Piazza, 2010). Some claim that non-symbolic magnitude abilities scaffold the acquisition of symbolic (Arabic number) magnitude abilities, which, in turn, support the acquisition of math ability (Dehaene, 2007, 2011; Piazza and Izard, 2009; Piazza, 2010; Siegler, 2016). Others, in contrast, claim that non-symbolic and symbolic magnitude abilities are independent of each other and exert independent effects on emerging math abilities (De Smedt et al., 2009; Holloway and Ansari, 2009; Maloney et al., 2010; Sasanguie et al., 2012a). The fact that research can be cited in support of both claims implies the developmental significance of the relationship between non-symbolic and symbolic magnitude representation and children’s math abilities is uncertain.

We suggest this uncertainty may be resolved by examining the relationship between patterns of differences in children’s non-symbolic and symbolic magnitude representation abilities and their associated math and cognitive abilities over time. Given math ability likely depends on both general/number-specific abilities (Jordan et al., 2013; Träff, 2013); it is important to model different general/number-specific relationships with different magnitude representation profiles. We further suggest that such an examination may reveal information about potentially different magnitude representation developmental pathways distinguishing between typical and atypical pathways that underpin different math outcomes (Reeve et al., 2018).

Findings from longitudinal research examining the relationships between non-symbolic, symbolic magnitude judgment and math abilities over time are mixed (Sasanguie et al., 2012a,b; Kolkman et al., 2013; Xenidou-Dervou et al., 2016). Desoete et al. (2012), for instance, assessed 5- to 6-year-olds on three occasions and found no correlation between non-symbolic and symbolic judgment accuracy. However, children’s non-symbolic and symbolic magnitude judgments were independently associated with math abilities. And 5- to 6-year-olds’ non-symbolic judgments predicted their calculation ability 1 year later and arithmetic fact retrieval 2 years later. Further, symbolic judgments were also associated with calculation. Vanbinst et al. (2015b) also found non-symbolic and symbolic magnitude abilities independently predicted 6-year old’s arithmetic accuracy and fact retrieval 1 year later; however, only symbolic magnitude ability predicted these outcomes 6 months later.

Others, in contrast, have found only symbolic magnitude abilities predict math abilities over time. Bartelet et al. (2014), for example, found 6-year-olds’ symbolic judgment efficiency (accuracy/RT) predicted arithmetic achievement 1 year later, whereas non-symbolic judgment did not. Nonetheless, they found correlations between non-symbolic and symbolic judgment, RT and efficiency measures. Similarly, Sasanguie et al. (2013) found 6- to 8-year olds’ symbolic, but not non-symbolic, judgment speed correlated with timed arithmetic and a standardized math test 1 year later. However, they did not find a correlation between symbolic and non-symbolic judgments.

While methodological factors (e.g., magnitude judgment measures, sample size and age) may contribute to the aforementioned differences in findings (Price et al., 2012; Xenidou-Dervou et al., 2016), they fail to account for all differences (Chen and Li, 2014; Fazio et al., 2014; Chew et al., 2016; Schneider et al., 2016). We suggest that the variability in both cross-sectional and longitudinal developmental magnitude representation research findings may reflect the use of variable-oriented analytic approaches for analyzing magnitude ability data, which focuses on the relations between variables (e.g., using aggregated data in correlations and regression models).

Aggregate data methods tend to assume (1) homogeneity with respect to how variables of interest influence each other, (2) deviations from the mean reflect measurement error and (3) within-age variability is noise (see Chew et al., 2016 for a discussion). In terms of developmental changes, aggregate methods assume “universal” patterns of change where the focus is a general model of normative (average) developmental changes. These methods, however, may mask the presence of different patterns of magnitude abilities and, ipso facto, the possibility that different development models of magnitude representation development reside within a general data distribution (Chew et al., 2016). Aggregating data is a dubious practice when within-age variability is systematically related to patterns of inter-individual development (Dowker, 2008; Bouwmeester and Verkoeijen, 2012; Reeve et al., 2012; Gray and Reeve, 2016; Paul and Reeve, 2016). Insofar as different patterns of non-symbolic–symbolic magnitude ability relationships can be identified, they would not be represented by a general model that would comprise a summary of the mixture patterns (Siegler, 1987; Bergman et al., 2003; Collins and Lanza, 2013; Paul and Reeve, 2016).

Some researchers have argued for person-centered analytic approach to better understand the significance of individual differences in patterns of early math cognition (Dowker, 2008; Reeve et al., 2012, 2018; Chew et al., 2016). A person-centered approach (1) rejects the assumption that the entire population is homogeneous with respect to how variables influence each other, and (2) attempt to identify individuals characterized by different patterns of associations that are similar within subgroups but are different between subgroups (Laursen and Hoff, 2006).

Latent class analysis is a statistical model-based approach for partitioning heterogeneity in a population by identifying a small group of homogenous latent subgroups embedded within a set of measures (Lanza and Cooper, 2016). Individuals are assigned to the subgroup for which the posterior probability of belonging to that subgroup is the highest; calculated as a function of the observed data and parameter estimates (Vermunt and Magidson, 2013b; Lanza and Cooper, 2016). Latent profile analysis can be extended to model longitudinal data, where transitions over time in latent subgroup membership are also estimated in the model (i.e., latent transition analysis, LTA). While subgroup membership is assumed to be stable in latent profile analysis (stable patterns of response characteristics), in LTA, individuals may change membership in latent profiles across time (see Hickendorff et al., 2018 for an analysis of the value of latent modeling for research on development and learning).

Chew et al. (2016) employed latent class analysis to determine whether different non-symbolic and symbolic magnitude (accuracy and judgment speed) ability profiles can be extracted from a general non-symbolic–symbolic magnitude ability distribution. They identified four different non-symbolic–symbolic magnitude ability profiles, three of which corresponded to the different pattern of findings identified in previous research (similarly good/bad non-symbolic–symbolic magnitude abilities; poor on symbolic relative to better non-symbolic magnitude abilities) (e.g., Halberda et al., 2008; Holloway and Ansari, 2009). These authors also found a previously unidentified fourth profile in which children displayed better symbolic magnitude ability relative to non-symbolic ability. Children assigned to this profile showed relatively superior symbolic magnitude judgment accuracy, albeit with longer response times. Moreover, the four identified magnitude abilities profiles were associated with different cognitive and math abilities. Chew et al. (2016) suggested that the different non-symbolic–symbolic magnitude/cognitive/math profiles reflect potentially different developmental patterns or models of math development. Children who possessed good or average non-symbolic and symbolic magnitude abilities showed relatively better visuospatial working memory, symbolic number access and math abilities. Children with poorer symbolic magnitude abilities, relative to non-symbolic abilities, performed poorer on a symbolic number access task and had poorer math abilities, compared to other magnitude profiles. Children in the fourth profile had relatively poorer visuospatial working memory and poorer math abilities. These findings highlight the fact that there is no single developmental model of magnitude representation underlying math abilities *per se*.

While this research highlight the value of latent profile analysis in potentially making sense of the heterogeneous distribution of non-symbolic and symbolic magnitude abilities and associated cognitive/math abilities in young children, the significance of their findings require explication in at least two ways. First, can Chew et al.’s (2016) findings be replicated? It has been argued that outcome of latent class modeling requires replication before claims can be made about the conceptual authenticity of identified profiles (Hickendorff et al., 2018). Second, since Chew et al.’s (2016) research was cross-sectional, we know little about the stability and/or change in the identified non-symbolic and symbolic magnitude ability profiles over time, or their relationship with cognitive and/or math abilities. The latter issue is particularly important. The degree to which deficits, delays or normal developmental profiles can be identified depends critically on longitudinal modeling (Reeve et al., 2012, 2018; Hickendorff et al., 2018). Nevertheless, both issues require answers before strong claims can be made about the developmental significance of different non-symbolic–symbolic magnitude ability profiles, especially with respect to the existence and significance of different magnitude representation developmental pathways.

### The Current Study

We employed latent class modeling of children’s non-symbolic and symbolic judgment responses, as well as of children’s cognitive and math abilities, to investigate the significance of the stability and/or change in different patterns of magnitude representation longitudinally. Our aim was to better understand the nature and significance of individual differences in patterns of math development which may be reflected as typical and atypical pathways (Dowker, 2008; Reeve et al., 2018). We used LTA to investigate 5- to 6-year-olds’ non-symbolic and symbolic magnitude judgment accuracy and RT signature patterns twice in 1 year. Our analytic approach is similar to the LTA modeling used by Reeve et al. (2018) who identified three distinct computation development trajectories, reflecting typical, delayed, and deficit pathways.

We assessed children’s VSWM and symbolic access ability since these abilities are often associated with magnitude representation and math abilities (De Smedt and Gilmore, 2011; Friso-Van Den Bos et al., 2013; Vanbinst et al., 2015b; Paul and Reeve, 2016). VSWM is thought to support numerical magnitude processing, predicated on the proposition that magnitude information is spatially organized (Dehaene, 1992; Dehaene and Cohen, 1997; Zorzi et al., 2002; Dehaene et al., 2003; de Hevia et al., 2008). The speed and accuracy naming numbers (Arabic digits) has been used to assess the ability to access number symbols information (i.e., symbolic number knowledge) which is often invoked as an explanation for differences in symbolic magnitude abilities and in turn, math abilities (Rousselle and Noël, 2007; Berteletti et al., 2010; De Smedt and Gilmore, 2011). Naming number ability is also a marker of symbolic access difficulty (Chew et al., 2016). We also included basic RT and a general intelligence measure since math ability is often associated with them (Kyttälä and Lehto, 2008; Geary, 2011; Luwel et al., 2013; Vanbinst et al., 2015b).

We assessed children’s single-digit addition and transcoding abilities to evaluate the relationship between profile membership and math abilities. We examined single-digit addition and transcoding (“reading” number strings) since they are considered important for later math abilities (Geary, 2000; OECD, 2012; Vanbinst et al., 2015a). In Australia single-digit addition is introduced to children from kindergarten onward and often used as an outcome measure (see Paul et al., 2018).

Based on Chew et al.’s (2016) findings, we anticipated identifying a profile that exhibited good, and one exhibiting average, non-symbolic and symbolic magnitude abilities (i.e., similar non-symbolic–symbolic magnitude abilities), and a profile that possessed better non-symbolic relative to symbolic magnitude ability. We also expected to identify a profile that exhibited better symbolic relative to non-symbolic magnitude ability. We expected children assigned to a good non-symbolic–symbolic magnitude ability profile to reflect a typical change pathway, and would exhibit good VSWM and naming number ability, and in turn, good single-digit addition and transcoding abilities across time. Insofar as other profiles reflect atypical change pathways, we expect children assigned to better non-symbolic relative to symbolic magnitude ability profile to possess poorer VSWM and those assigned to better symbolic relative to non-symbolic magnitude ability profile to possess poorer naming number ability. Children displaying relatively poorer non-symbolic and/or poorer symbolic magnitude abilities would also perform poorer on single-digit addition and transcoding. While some children may move from one profile to another over time, we do not expect a child who belonged to a better performing profile (relative to other children) would move to a poorer profile over time.

## Materials and Methods

### Participants

One-hundred-nine children (55% females) participated, comprising 48 Kindergarten (*M* = 5.8 years, *SD* = 2.8 months) and 61 Year 1 (*M* = 6.8 years, *SD* = 3.6 months) children at initial assessment. All spoke English, had normal or corrected-to-normal vision and had no identified learning difficulties. The study was conducted with the approval of, and in accordance with, the authors’ University’s human research ethics committee. The parents of children provided informed consent for their children to participate in the study.

### Procedure

All children individually completed non-symbolic and symbolic magnitude judgments, naming numbers, single-digit addition, reading numbers, Corsi Blocks Backward (VSWM), Raven’s Colored Progressive Matrices (non-verbal IQ) and basic RT tasks on the first assessment. Approximately 1 year later, they completed the non-symbolic and symbolic judgment tasks, and the single-digit addition and reading numbers tasks. Tasks were completed in short sessions over 3 days (non-symbolic and symbolic tasks were completed on separate days to avoid inter-task priming effects). Except for the non-verbal IQ and VSWM tasks, stimuli were presented on a 15″ screen laptop computer running E-Prime software (version 2.0). The screen was at eye-level, approximately 40 cm in front of children. A fixation cross appeared in the center of the screen prior to a target stimulus appearing. Except for non-symbolic and symbolic tasks, in which response time was capped at 5,000 ms, stimuli remained on the screen until a response was made.

### Non-symbolic and Symbolic Judgment Tasks

In the non-symbolic judgment task, two sets of blue squares separated by a central vertical line appeared on the screen (Chew et al., 2016). Children selected the set that had the most squares by pressing the corresponding right shift key or the left shift key. The task comprised 72 trials with judgment combinations of all quantities between one and nine blue squares, except ties (e.g., 9 and 9). The ratios for each trial (i.e., smaller number/larger number) were divided into eight ratios: 0.1-0.19; 0.2-0.29… up to 0.8-0.89. Stimuli were presented in a fixed random order, with the larger set appearing on the left- and right-hand sides of the screen equally. To reduce possible reliance on perceptual cues for judgments, individual square sizes and total area were systematically varied across trials (total area was the same for both sets within trials) (Dehaene et al., 2005). We analyzed two indices (mean accuracy and median RT) (Bartelet et al., 2014; Ratcliff et al., 2015; Schneider et al., 2016), both of which are associated with math ability (De Smedt et al., 2013).

The symbolic judgment task was procedurally identical to the non-symbolic task, except black Arabic digits were presented on white background (60-point font size).

### Number Naming

Children named digits between 1 and 9; each digit was presented three times in separate blocks of trials (*n* = 27 trials overall). The interviewer pressed a response key following each response and recorded responses verbatim (the interviewer could not see the computer screen). Median RT was used for analysis since children made few errors.

### Single-Digit Addition

Children completed 30 two-term addition problems, following two practice trials. They were instructed to answer problems as quickly and as accurately as possible. Addends comprised combinations of all digits between “2” and “7” (excluding tied pairs: e.g., 2 + 2), in both orders (e.g., 2 + 7 and 7 + 2). Single-digit addition problems are widely used as a measure of early computation ability (Bailey et al., 2012; Paul and Reeve, 2016).

### Transcoding: Reading Multi-Digit Numbers

Children read 30 two to four digit numbers displayed on the computer screen (i.e., 11, 12, 14, 16, 17, 19, 28, 35, 47, 52, 73, 94, 105, 162, 207, 435, 574, 809, 1002, 2584, 3201, 4783, 6057, 9236, 10006, 26103, 50316, 46927, 60935, and 79768). The numbers were presented in the same randomized order for all children.

### Corsi Blocks Backward (VSWM)

The interviewer tapped a sequence of blocks in a pre-specified order and children attempt to repeat the tap sequences in reverse order (Kessels et al., 2000). Children were ensured that they understood the task in preceding practice trials. Testing ceased after two failed trials. The VSWM span comprised the average of the longest correct reverse block tap sequences.

### Raven’s Colored Progressive Matrices (Non-verbal IQ)

RCPM was administered following manual instructions and responses scored using published age norms (Raven et al., 1995; Cotton et al., 2005).

### Basic RT

The task comprised nine trials. Children pressed a computer key as quickly as possible when a black dot appeared on the screen approximately 500 ms later after a central fixation point.

### Analytic Approach

We used LTA to identify distinct profiles (i.e., subgroups) of children who share similar non-symbolic–symbolic magnitude judgment accuracy/RT response patterns, and examined changes in profile membership over time (Latent GOLD 5.1; Vermunt and Magidson, 2015). Similar to latent profile analysis, we rely on a set of criteria for selecting the optimal model solution (Trezise and Reeve, 2014; Chew et al., 2016). Goodness-of-fit statistics (e.g., Bayesian information criterion) weigh the fit of the models relative to the number of parameters, with a lower value indicating a better fitting model to the data (Vermunt and Magidson, 2013a). Entropy, which range from 0 to 1, assess how well the subgroups are classified and values greater than 0.8 are considered to have high entropy which implies better classification (Clark and Muthen, 2009). The theoretical relevance and usefulness of the latent profiles were also considered (Muthen and Muthen, 2000). Models were fit using 200 random starting sets and 500 replications to ensure that model convergence could be replicated.

The LTA model includes three types of parameters. It yields the conditional response probabilities that describe response patterns conditional on latent subgroup membership. For example, a profile with a relatively high probability of high accuracy and RT on non-symbolic/symbolic judgments can be interpreted as showing good non-symbolic–symbolic magnitude abilities. The model also yields class probabilities, which describe the size of each latent subgroup at each time point (i.e., relative frequency of class membership) and a matrix of transition probabilities (i.e., conditional probabilities describing the probability of being in a given subgroup at time = *t*, conditional on the subgroup at time = *t -* 1) which describes how children transition from Time 1 to Time 2 in non-symbolic–symbolic magnitude ability profiles. Measurement invariance was modeled (i.e., conditional response probabilities are the same across the two time points), following from previous work (Chew et al., 2016) and initial examination showing consistency in profiles at both time points. The same number and type of classes occur at both time points allowing a straightforward interpretation since the meanings of the profiles are the same across time.

The following covariates were included in the model as predictors of latent profiles at Time 1 and 2, as well as predictors of transitions in profile membership between Time 1 and 2: VSWM, naming numbers, basic RT, non-verbal IQ and grade. When covariates are included in the LTA model (i.e., in a 1-step model), current profile membership (i.e., described by transitional probabilities) is predicted by both profile membership at the previous time point and the value of the covariates. Class profiles, class sizes and transition probabilities may change as a result.

A three-step estimation procedure (Vermunt and Magidson, 2015) was separately conducted for the LTA model where the association between the predictor variables and assigned membership are examined at time points and the underlying statistical model is analogous to a multinomial regression logistic regression. The step-three modeling approach allows for the correction of classification errors obtained when assigning profile memberships (maximum-likelihood adjustment method is used to correct for classification errors) at the particular time points—a failure to account for classification errors can lead to an underestimation of the relationship between profile membership and other variables (Bakk et al., 2013). This estimation approach is desirable in the LTA context because the 1-step model approach (i.e., covariates included in model) has the drawback that covariate values at one point in time affects the definition of the latent class variable at another point in time. Similarly, SDA and transcoding abilities (treated here as dependent variables) were regressed on the latent profile membership at Time 1 and 2.

## Results

As expected, non-symbolic and symbolic magnitude RT and error rates increased with increasing ratios and decreased with increasing grade (descriptive statistics are reported in Supplementary Material). Means and standard deviations for all measures as a function of grade are reported in Table 1. Zero-order correlations among measures are reported in Table 2 which shows significant correlations between children’s non-symbolic–symbolic magnitude judgments, and SDA problem solving and transcoding abilities. Non-symbolic and symbolic accuracy/RTs were correlated at Time 1 and Time 2. Similarly, non-symbolic and symbolic accuracy and RT at Time 1 correlated with the same measures at Time 2 (except non-symbolic RT).

**Table 1:** NSM and SM measures, cognitive factors, and math abilities as a function of grade.

	Grade			
	Kindergarten	Year 1	Year 1	Year 2
		
	*M* (*SD*) at Time 1		*M* (*SD*) at Time 2	
NSM correct	0.79 (0.14)	0.87 (0.08)	0.92 (0.06)	0.94 (0.05)
NSM RT	1479.53 (387.84)	1287.22 (317.95)	1092.89 (231.06)	958.08 (271.72)
SM correct	0.82 (0.16)	0.89 (0.08)	0.91 (0.05)	0.93 (0.05)
SM RT	1344.43 (416.02)	1183.39 (273.18)	1148.64 (199.7)	905.47 (255.1)
Transcoding	42.53 (23.87)	80.94 (15.09)	75 (18.79)	96.65 (6.36)
SDA correct	42.57 (35.78)	91.04 (9.9)	86.67 (19.51)	95.03 (8.25)
VSWM	3.25 (0.64)	3.6 (0.85)	-	-
Naming Numbers	956.56 (242.05)	840.97 (190.59)	-	-
RCPM	80.73 (22)	83.52 (18.33)	-	-
Basic RT	632.70 (82.46)	589.47 (88.47)	-	-

*NSM, non-symbolic magnitude; SM, symbolic magnitude; M, means except median for NSM RT, SM RT, naming numbers, and basic RT.*

**Table 2:** Zero-order correlations among NSM-SM measures, cognitive factors, and math abilities.

			Time 1				Time 2								Time 1		Time 2	
			1	2	3	4	5	6	7	8	9	10	11	12	13	14	15	16
Time 1	1. NSM correct	1															
	2. SM correct	0.44^**^	1														
	3. NSM RT	-0.26^**^	-0.02	1													
	4. SM RT	-0.18	0.04	0.54^**^	1												
Time 2	5. NSM correct	0.29^**^	0.28^**^	-0.17	-0.1	1											
	6. SM correct	0.2^*^	0.45^**^	-0.06	0.04	0.31^**^	1										
	7. NSM RT	-0.3^**^	-0.23^*^	0.17	0.13	-0.41^**^	-0.13	1									
	8. SM RT	-0.2^*^	-0.37^**^	0.24^*^	0.3^**^	-0.34^**^	-0.24^*^	0.64^**^	1								
	9. VSWM	0.14	0.35^**^	-0.11	-0.09	0.26^**^	0.18	-0.2^*^	-0.2^*^	1							
	10. NN	-0.11	-0.18	0.18	0.08	-0.24^*^	-0.12	0.4^**^	0.4^**^	-0.04	1						
	11. RCPM	0.1	0.16	0.04	-0.02	0.15	0.13	-0.09	-0.18	0.19^*^	-0.16	1					
	12. Basic RT	-0.26^**^	-0.05	0.24^*^	0.28^**^	-0.06	0.08	0.13	0.14	-0.1	0.21^*^	0.06	1				
Time 1	13. SDA correct	0.31^**^	0.42^**^	-0.21^*^	-0.15	0.17	0.19^*^	-0.31^**^	-0.33^**^	0.35^**^	-0.33^**^	0.16	-0.16	1			
	14. Transcoding	0.21^*^	0.42^**^	-0.12	-0.2^*^	0.19	0.32^**^	-0.26^**^	-0.43^**^	0.28^**^	-0.43^**^	0.11	-0.18	0.72^**^	1		
Time 2	15. SDA correct	0.22^*^	0.42^**^	0.03	-0.07	0.31^**^	0.4^**^	-0.33^**^	-0.33^**^	0.25^**^	-0.34^**^	0.25^**^	-0.11	0.28^**^	0.42^**^	1	
	16. Transcoding	0.37^**^	0.5^**^	-0.14	-0.2^*^	0.22^*^	0.33^**^	-0.38^**^	-0.47^**^	0.31^**^	-0.42^**^	0.19^*^	-0.2^*^	0.68^**^	0.73^**^	0.49^**^	1

*NSM, non-symbolic magnitude; SM, symbolic magnitude; NN, naming numbers RT. ^∗^p < 0.05, ^∗∗^p < 0.01.*

### Assessing Model Fit

Models comprising one to six latent profiles were estimated from non-symbolic and symbolic magnitude RT and accuracy at Time 1 and 2. Goodness-of-fit indices for each model are reported in Table 3: a four-profile solution was selected as the best fitting model. A three and five profile solution were also considered; the three-profile solution was not optimal when analyzed at Time 1 and 2 separately while the five profile had low interpretability (e.g., the fifth profile had a small number of children who appeared similar to another profile). The four-profile model was selected on the basis of fit, previous research (Chew et al., 2016) and conceptual interpretability. Model selection was supported by a high entropy value (i.e., above 0.8), indicating good classification of individuals into latent profiles.

**Table 3:** Fit information for the latent transition analysis model.

*N* Profiles	*N* par	*Log likelihood*	*aBIC*	*AIC3*	*CAIC*	Entropy R-squared
1	8	-2781.95	5581.62	5587.89	5609.42	1
2	19	-2536.91	5115.91	5130.81	5181.95	0.80
3	32	-2453.60	4978.08	5003.18	5089.31	0.83
**4**	**47**	**-2420.46**	**4945.05**	**4981.92**	**5108.41**	**0.83**
5	64	-2396.88	4935.55	4985.75	5158.00	0.82
6	83	-2380.38	4944.65	5009.75	5233.14	0.85

*Bold values indicate best fitting model. N Profiles, number of latent profiles; N par, number of parameters in the model; aBIC, adjusted Bayesian information criteria; AIC3, Akaike’s information criterion with 3 as penalizing factor; CAIC, consistent AIC.*

### Non-symbolic–Symbolic Magnitude Ability Profiles

**FIGURE 1:**
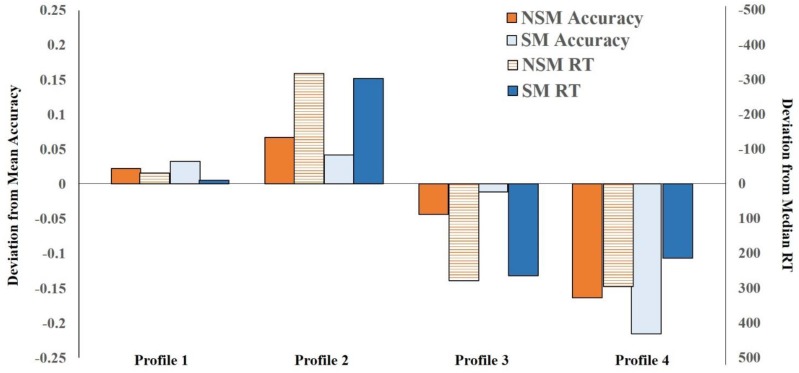
Deviations from non-symbolic magnitude (NM) and symbolic magnitude (SM) overall mean proportion accuracy (left *y*-axis) and median RT (right *y*-axis) as a function of profile membership from Time 1 to Time 2.

Deviations from overall mean proportion accuracy (non-symbolic = 0.88; symbolic = 0.89) and median RT (non-symbolic = 1236.54 ms; symbolic = 1179.84 ms) for the four profiles across Time 1 and 2 are presented in Figure 1. Labels corresponding to the relative non-symbolic and symbolic magnitude abilities were assigned to each profile. (Note, the numbers attached to the profiles–i.e., profile 1, 2, 3, 4–are convenient labels and not a statement about the ordinal position of the profiles.) Profile 1 comprised children who displayed average accuracy and speed on both non-symbolic and symbolic judgments (i.e., relatively close to the overall mean/median). They were characterized by “average non-symbolic–symbolic magnitude abilities.” Profile 2 included children who were relatively highly accurate and fast on both non-symbolic and symbolic judgments, and they were characterized by “good non-symbolic–symbolic magnitude abilities.” Children in Profile 3 were relatively average on symbolic judgments but much less accurate on non-symbolic judgments relative to non-symbolic. They also exhibited relatively long response speed on both non-symbolic and symbolic judgments. Hence, they were characterized by “better symbolic abilities relative to non-symbolic abilities.” Children in Profile 4 were less accurate and slower on both non-symbolic and symbolic judgments relative to other children. However, they were more accurate on non-symbolic relative to symbolic judgments. They were characterized by “better non-symbolic abilities relative to symbolic abilities.” One-way ANOVAs and Bonferroni-corrected *post hoc* comparisons showed profiles differed from each other in non-symbolic and symbolic magnitude, accuracy and RT (details are reported in the Supplementary Material).

### Change/Stability Patterns inNon-symbolic–Symbolic MagnitudeProfiles Over Time

Table 4 presents the transition probabilities, which reflect the probability of a child transitioning to a particular profile at Time 2, conditional on their profile membership at Time 1. These parameters describe the patterns of change in non-symbolic–symbolic magnitude abilities across time. (The probabilities may also be considered to reflect the proportion of each profile at Time 1 that transitioned into particular profiles at Time 2.) Diagonal values indicate the proportion of children who remained in the same profile at both times. Off-diagonal values indicate the proportion of children in a particular profile at Time 1, who move into another profile at Time 2. Results indicate that membership to Profile 2 was stable; 96% (*n* = 4) of children who were in Profile 2 at Time 1, remained in Profile 2 at Time 2. In other words, it is very unlikely (low probability) that children in this Profile would move into any of the other Profiles.

**Table 4:** Latent transition probabilities based on latent transitional analysis model for NSM and SM.

	Time 2 status				
Time 1 status	Profile 1	Profile 2	Profile 3	Profile 4	*N*
Profile 1	**0.0196**	0.9727	0.0059	0.0017	39
Profile 2	0.0138	**0.9578**	0.0144	0.0139	4
Profile 3	0.6745	0.2898	**0.0342**	0.0015	46
Profile 4	0.4770	0.1041	0.4153	**0.0036**	20
*N*	42	57	10	0	109

*Bold values indicate the proportion of children who remained in the same profile at T1 and T2. NSM, non-symbolic magnitude; SM, symbolic magnitude.*

Children who were in Profile 1 at Time 1 had a high likelihood (0.97 probability) of moving into Profile 2 at Time 2. That is, 97% (*n* = 38) of the Profile 1 children at Time 1 moved into Profile 2 at Time 2. It was unlikely that children in this Profile moved into other profiles. Sixty-seven percent of children (*n* = 31) who were in Profile 3 at Time 1 moved into Profile 1 at Time 2; followed by Profile 2 (29%; *n* = 13). Finally, of the children in Profile 4 at Time 1, 48% (*n* = 10) moved into Profile 1 and 42% (*n* = 8) into Profile 3 at Time 2. It was rare (10%; *n* = 2) that they moved into Profile 2 at Time 2. Of note, all children in Profile 4 at Time 1 moved into other profiles at Time 2 and no children moved into this profile at Time 2 (i.e., there were no children in Profile 4 at Time 2).

In sum, most children change in profile membership over time, except those in Profile 2 who exhibited stability. Specifically, children moved to a better non-symbolic–symbolic magnitude ability profile over time. Frequencies in profiles as a function of grade at Time 1 and 2 are presented in Table 5.

**Table 5:** Frequencies in non-symbolic–symbolic magnitude profiles as a function of grade at Time 1 versus Time 2.

	Time 1		Time 2	
	Kindergarten	Year 1	Year 1	Year 2
Profile 1	8	31	26	16
Profile 2	0	4	15	42
Profile 3	24	22	7	3
Profile 4	16	4	0	0

### Predicting Non-symbolic–Symbolic Magnitude Ability Profiles

To examine whether cognitive measures predicted transitions in non-symbolic–symbolic magnitude profiles from Time 1 to 2, five predictors were included in the LTA model. (Grade was included to examine possible age-related effects on profile membership across time.) The overall model showed VSWM (*Wald* = 6.56, *p* = 0.88), non-verbal IQ (*Wald* = 7.16, *p* = 0.85), basic RT (*Wald* = 9.22, *p* = 0.69), naming number ability (*Wald* = 6.77, *p* = 0.87) and grade (*Wald* = 4.69, *p* = 0.97) did not reach statistical significance. Next, we used the three-step procedure to determine whether initial cognitive measures/age (Time 1) predicted profile membership at both time points. We examined the standardized regression coefficients (z-scores) and Wald statistics for each measure predicting profile memberships in a multivariate model that accounts for classification errors (see Table 6). The z-scores show the predictive effect of factors for profiles while taking into account other variables in the model. Findings show VSWM and naming numbers independently predict non-symbolic–symbolic magnitude profiles at both Time 1 and 2, whereas non-verbal IQ and basic RT did not. Age was only associated with profile membership at Time 1.

**Table 6:** Time 1 covariates predicting NSM-SM profile memberships at Time 1 and Time 2.

		NSM–SM profiles				
			Profile 1	Profile 2	Profile 3	Profile 4
*N* = 160	*Wald*	*P*	*z-score*	*z-score*	*z-score*	*z-score*
**Time 1**						
**Age**	**11.4**	**0.009**	**0.33**	2.11^*^	-2.18^*^	-2.76^**^
**VSWM**	**10.68**	**0.014**	**-0.37**	2.06^*^	**-0.7**	-3.23^**^
**Naming number**	**9.72**	**0.021**	**0.94**	-2.74^**^	2.44^*^	3.09^**^
RCPM	7.32	0.062	2.38	-2.21	0.053	1.25
Basic RT	6.05	0.11	2.01	-2.14	2.28	0.98
**Time 2**						
**Age**	**6.81**	**0.033**	**-1.53**	**1.73**	**-0.12**	–
**VSWM**	**7.02**	**0.03**	**-0.26**	2.48^*^	**-1.32**	–
**Naming number**	**12.42**	**0.002**	**0.41**	-3.45^**^	2.96^**^	–
RCPM	2.01	0.37	-1.07	1.04	0.15	–
Basic RT	0.34	0.84	0.52	0.53	-0.58	–

*Bold values indicate significant predictors. NSM, non-symbolic magnitude; SM, symbolic magnitude. ^∗^p < 0.05, ^∗∗^p < 0.01.*

At Time 1, an increase in age was associated with an increased likelihood of belonging to Profile 2 (*B* = 0.24, *SE* = 0.11, *z* = 2.11) and a reduced likelihood of belonging to Profiles 3 (*B* = -0.1, *SE* = 0.04, *z* = -2.18) and 4 (*B* = -0.15, *SE* = 0.06, *z* = -2.76). An increase in VSWM was associated with an increased likelihood of belonging to Profile 2 (*B* = 2.25, *SE* = 1.09, *z* = 2.06) and conversely, a reduced likelihood of belonging to Profile 4 (*B* = -1.8, *SE* = 0.56, *z* = -3.23). A poorer naming number ability (i.e., longer naming number RT) was associated with a reduced likelihood of belonging to Profile 2 (*B* = -0.01, *SE* = 0.004, *z* = -2.74) and a greater likelihood of belonging to Profiles 3 (*B* = 0.004, *SE* = 0.002, *z* = 2.44) and 4 (*B* = 0.005, *SE* = 0.002, *z* = 3.09).

At Time 2, an increase in VSWM was associated with a greater likelihood of belonging to Profile 2 (*B* = 0.74, *SE* = 0.3, *z* = 2.48). A poorer naming number ability was associated with a reduced likelihood of belonging to Profile 2 (*B* = -0.004, *SE* = 0.001, *z* = -3.45) and a greater likelihood of belonging to Profile 3 (*B* = 0.004, *SE* = 0.001, *z* = 2.96).

Overall, at Time 1, children in Profile 2 were more likely to be older, and conversely children in Profiles 3 and 4 were more likely to be younger. However, at Time 2, age was no longer associated with profile membership. At Time 1, higher VSWM and naming number ability characterized children in Profile 2, whereas poorer VSWM and naming number ability characterized children in Profile 4. Poorer naming number ability also characterized children in Profile 3. At Time 2, higher VSWM and naming number ability remained characteristic of children in Profile 2, whereas poorer naming number ability remained characteristic of children in Profile 3.

### Non-symbolic–Symbolic Magnitude Profiles Predicting Math Abilities

SDA and transcoding were regressed on profile membership at Time 1 and 2 while accounting for classification errors using the three-step procedure. Accuracy reading teen, two digit, three digit and four digit numbers showed reasonably good internal consistency (Cronbach alpha = 0.77) and hence, accuracy was summed across these digit strings. The standardized regression coefficients (z-scores) and Wald statistics for each dependent variable predicted by profile membership for both time points are reported in Table 7. SDA correctness and transcoding were significantly associated with profile membership at Time 1 and 2. At Time 1, an increase in SDA accuracy was associated with belonging to Profiles 1 and 2 and, conversely, a reduced likelihood of belonging to Profile 4. An increase in transcoding ability was associated with belonging to Profiles 1 and 2 and, conversely, a reduced likelihood of belonging to Profiles 3 and 4. At Time 2, an increase in SDA accuracy and transcoding was associated with belonging to Profile 2.

**Table 7:** Significant effects in three-step latent profile model with dependent variables at Time 1 and Time 2.

		NSM–SM profiles				
*N* = 160	*Wald*	*p*	Profile 1	Profile 2	Profile 3	Profile 4
**Time 1**						
SDA	60.16	<0.001	*B* = 18.51,	*B* = 22.09,	*B* = -7.59,	*B* = -33.01,
			*SE* = 3.81,	*SE* = 3.49,	*SE* = 5.13,	*SE* = 7.17,
			*z* = 4.86^∗∗^	*z* = 6.34^∗∗^	*z* = -1.48	*z* = -4.60^∗∗^
Transcoding	44.47	<0.001	*B* = 16.52,	*B* = 14.54,	*B* = -8.77,	*B* = -22.29,
			*SE* = 4.07,	*SE* = 5.25,	*SE* = 3.72,	*SE* = 4.89,
			*z* = 4.06^∗∗^	*z* = 2.77^∗^	*z* = -2.36^∗^	*z* = -4.56^∗∗^
**Time 2**						
SDA	13.62	0.001	*B* = 3.18,	*B* = 10.49,	*B* = -13.67,	–
			*SE* = 4.38,	*SE* = 3.63,	*SE* = 7.52,	
			*z* = 0.73	*z* = 2.89^∗∗^	*z* = -1.82	
Transcoding	38.81	<0.001	*B* = -3.06,	*B* = 14.37,	*B* = -11.31,	–
			*SE* = 3.75,	*SE* = 3.09,	*SE* = 6.06,	
			*z* = -0.82	*z* = 4.64^∗∗^	*z* = -1.87	

*NSM, non-symbolic magnitude; SM, symbolic magnitude. ^∗^p < 0.05, ^∗∗^p < 0.01.*

## Discussion

The purpose of the present study was to assess the degree to which (1) the different non-symbolic–symbolic magnitude representation profiles identified in a previous study would be re-identified, and (2) profiles remained stable or changed over time. The aim was to determine whether stability/change in profiles were related to children’s cognitive and math abilities. Of interest was whether we could identify different magnitude representation pathways that distinguish typical and atypical models of math development. Four findings are of note. First, the current study replicates Chew et al. (2016) by showing that four meaningfully different non-symbolic–symbolic magnitude ability profiles can be extracted from a general non-symbolic–symbolic magnitude ability distribution. Second, the change/stability in profiles across time suggests different magnitude representation developmental pathways can be identified. Third, VSWM and naming number abilities were associated with profile membership at Time 1 and Time 2; however, they did not predict stability/change in profile membership over time. Fourth, non-symbolic–symbolic magnitude ability profiles were differentially associated with math abilities at Time 1 and Time 2.

### Non-symbolic–Symbolic Magnitude Ability Profiles

The mean accuracy and median RTs for both non-symbolic and symbolic magnitude judgments of children in Profile 1 were close to the average mean accuracy and median RTs for the entire sample. Children in Profile 2 were more accurate and faster making both non-symbolic and symbolic magnitude judgments, relative to children in other profiles. The non-symbolic–symbolic magnitude judgment response patterns of children in Profiles 1 and 2 are consistent with claims made for an association between non-symbolic and symbolic magnitude abilities (Piazza et al., 2010; Dehaene, 2011; Feigenson et al., 2013).

Children in Profile 3 were more accurate in their symbolic magnitude judgments, relative to their non-symbolic judgments, but compared to Profiles 1 and 2, they were also relatively slower in making non-symbolic and symbolic magnitude judgments. This pattern of non-symbolic–symbolic magnitude judgment replicates Chew et al.’s (2016) findings. They suggested that symbolic abilities can be supported by rote practice. Some children may learn by rote recall and complete the symbolic judgments with some success but doing so requires more effort (i.e., longer RT and hence, possibly less efficient).

Children in Profile 4 were less accurate and slower making non-symbolic and symbolic magnitude judgments, compared to children in the other three profiles. However, they were more accurate making non-symbolic judgments compared to symbolic judgments, which is consistent with claims that symbolic magnitude abilities can be independent of non-symbolic abilities (i.e., better non-symbolic abilities relative to symbolic abilities–see Rousselle and Noël, 2007; Holloway and Ansari, 2009; Sasanguie et al., 2014).

### Change/Stability Patterns in Non-symbolic–Symbolic Magnitude Ability Profiles

Most children’s non-symbolic and symbolic magnitude abilities changed across time. The general “movement” pattern was from a less accurate and slower non-symbolic–symbolic magnitude profile to a more accurate and faster ability profile. No child moved from a better ability profile at Time 1 to a poorer one at Time 2. Only a small group of children (96%; *n* = 4) were stable across time (i.e., remained in Profile 2 across time); this stability suggests a consistency in good non-symbolic–symbolic magnitude abilities across time. Almost all children (97%; *n* = 38) moved from Profile 1 to Profile 2 at Time 2. While some children (29%; *n* = 13) moved from Profile 3 to Profile 2 at Time 2, the majority (67%; *n* = 31) moved to Profile 1. Similar proportions of children moved from Profile 4 to Profiles 1 (48%; *n* = 10) and 3 (42%; *n* = 8) at Time 2. Children in this profile rarely moved to Profile 2 at Time 2 (10%; *n* = 2).

The change in profile membership from Profile 1 (average non-symbolic–symbolic magnitude ability) to Profile 2 (good non-symbolic–symbolic magnitude ability) at Time 2 could be regarded as representing an expected change pathway. However, other changes in profile membership over time (e.g., Profile 4 to Profile 3) suggest that there may be other, possibly less optimal, change pathways to consider (i.e., atypical pathway). While there may be more than one non-symbolic–symbolic magnitude developmental pathway, they may represent different routes to competency (equifinality) or indicators of difficulties over time. For instance, the movement of some Profile 4 children (relatively better non-symbolic to symbolic magnitude ability) to Profile 3 (relatively better symbolic to non-symbolic magnitude ability) at Time 2 suggests that some children continue to develop symbolic abilities, separate from non-symbolic abilities. These children may represent relatively poorer developmental change (possibly reflecting a math delay or a deficit) in that they are not transitioning into a profile better on both symbolic and non-symbolic magnitude abilities. The movement of other Profile 4 children to Profile 1 at Time 2 suggests some children do continue to improve in both non-symbolic and symbolic magnitude abilities – consistent with claims that non-symbolic magnitude ability supports symbolic ability.

The question of multiple developmental routes to equifinality or even math difficulties cannot be answered with only two time points, 1 year apart. However, current findings caution against the assumption of one general developmental pathway. Since different non-symbolic–symbolic magnitude ability profiles exist, it would be inappropriate to represent these two magnitude representation ability by a general model that reflects normative developmental changes (i.e., variable-centered analytical approaches). Using LTA allowed us to examine individual differences in patterns of change over time in which more than one developmental trajectories can systematically differ across individuals.

### Cognitive Factors/Age and Non-symbolic–Symbolic Magnitude Ability Profiles

While older children were likely to belong to Profile 2 and younger children were more were likely to belong to Profiles 3 and 4; grade only partially overlapped with profile membership. Children from both grades were represented in all profiles at Time 1 (except no kindergartener children were assigned to Profile 2). At Time 2, age was not associated with profile membership. Using LTA to characterize age variability allowed us to sidestep the assumption of age as proxy for development and, examine how age is related to the magnitude profiles a posteriori and how the cognitive factors related to profiles after taking age into account. Our findings are consistent with recent studies that caution against focusing on age differences which may mask meaningful profiles of competence (Gray and Reeve, 2014; Paul and Reeve, 2016).

VSWM, naming number ability, non-verbal IQ, basic RT nor age predicted changes in non-symbolic–symbolic magnitude ability profile membership across time. However, VSWM and naming number abilities at Time 1 were associated with profile memberships at both time points. This finding is consistent with studies that have found a link between VSWM/naming number abilities and math ability in young children (De Smedt et al., 2009; Berteletti et al., 2010; Mammarella et al., 2010; Vanbinst et al., 2015b).

At Time 1, poorer VSWM was associated with Profile 4; and poorer naming number ability was associated with Profiles 3 and 4. Conversely, good VSWM and naming number ability were associated with Profile 2. Similarly, at Time 2, good VSWM and naming number ability were associated with Profile 2 while poorer naming number ability was associated with Profile 3. The findings are consistent with claims that the ability to access symbolic numerical information is a number-specific cognitive factor for children who show poor symbolic magnitude abilities (i.e., Profile 4) (Rousselle and Noël, 2007; Holloway and Ansari, 2009).

Of interest, poorer naming number ability is also characteristic of children who displayed better symbolic magnitude abilities relative to non-symbolic abilities (Profile 3) at both time points. While children in Profile 3 completed symbolic magnitude judgments accurately, they took longer in making judgments. Nevertheless, their basic RT was not significantly different to other children. Naming numbers may be a useful marker of children’s ability to efficiently access symbolic magnitude information (Vanbinst et al., 2015b). Indeed, good naming number ability predicted good non-symbolic–symbolic magnitude abilities (Profile 2) at first test occasion and 1 year later.

Magnitude information is argued to be encoded spatially and VSWM is implicated in the representation and manipulation of numerical magnitudes more generally (Zorzi et al., 2002; Dehaene and Brannon, 2011; de Hevia, 2016). Although poorer VSWM was associated with Profile 4 children (i.e., relatively better non-symbolic to symbolic magnitude abilities), they were also children who displayed the weakest non-symbolic abilities at initial assessment (all moved out of the profile at Time 2). On the other hand, good VSWM predicted good non-symbolic–symbolic magnitude abilities (i.e., Profile 2) at both time points. This finding suggest that VSWM capacity may underpin non-symbolic magnitude abilities and, in turn, symbolic magnitude development and math abilities for some children.

### Non-symbolic–Symbolic Magnitude Ability Profiles and Math Abilities

Non-symbolic–symbolic magnitude ability profiles were associated with SDA and transcoding at both Time 1 and 2. This is consistent with research which has found a link between non-symbolic and/or symbolic magnitude abilities and math abilities (De Smedt et al., 2009; Bugden and Ansari, 2011; Mazzocco et al., 2011; Xenidou-Dervou et al., 2016). At Time 1, children with good/average non-symbolic–symbolic magnitude abilities relative to other children (i.e., Profiles 1 and 2) were associated with better SDA problem-solving and transcoding. In contrast, children with better non-symbolic abilities relative to symbolic abilities (Profile 4), was associated with poorer SDA problem-solving and transcoding. This finding is consistent with studies that show poorer symbolic magnitude judgment (and symbolic number access) are associated with poorer math abilities (Rousselle and Noël, 2007; De Smedt and Gilmore, 2011).

Children with better symbolic abilities, relative to non-symbolic abilities (Profile 3), possessed poorer transcoding skills—a finding that runs contrary to claims that symbolic magnitude abilities alone are associated with math abilities (Holloway and Ansari, 2009; Vanbinst et al., 2015a). Children in Profile 3 were only poorer on transcoding but not SDA accuracy. It is possible that some children are able to deploy compensatory strategies (e.g., finger-counting) to solve SDA problems and rely less on direct retrieval of arithmetic facts (Geary and Hoard, 2005).

At Time 2, only children with good non-symbolic–symbolic magnitude abilities (i.e., Profile 2) possessed better SDA problem-solving and transcoding. These findings suggest that non-symbolic magnitude abilities are important for symbolic magnitude abilities, and ipso facto, math abilities (i.e., good non-symbolic–symbolic magnitude abilities in Profile 2 at both time points).

### Implications and Directions for Future Research

The different change patterns of non-symbolic–symbolic magnitude abilities appear to represent different developmental change pathways. Current findings illustrate a multivariate framework in which different magnitude representation developmental pathways are underpinned by different cognitive factors (VSWM and naming numbers ability) that contribute to differences in math development. Insofar as different magnitude representation change pathways exist, they may reflect typical and atypical models of math development. Nevertheless, since the current study only investigated magnitude abilities over 1 year, caution should be exercised in extrapolating beyond this time period. We are unable to specify what happens to profile membership as children age: it is possible that the profiles will converge on a single magnitude ability competency (i.e., equifinality). It is also possible that differences in profile trajectories will remain separate or diverge further over time. Indeed, an explicit characterization of profile membership over time might help distinguish between delays, differences, deficits in math development. We suggest two of the change pathways (e.g., children transitioning from Profiles 4 to 3 and/or remaining in Profile 3) may well represent an early risk marker for math difficulties (delay and/or deficit). These issues are matter for future research, however. Nonetheless, it should be noted that VSWM and naming numbers ability are important correlates of a typical (and optimal) magnitude representation developmental pathway, and ipso facto, good math abilities.

Our research highlights the value of using LTA for examining data in which more than one developmental trajectories are hypothesized (Lanza and Cooper, 2016). Longitudinal analyses tend to focus on analytical techniques (e.g., correlations, regressions and structural equation models) where the same over time estimates are applied to samples (e.g., Libertus et al., 2013; Vanbinst et al., 2015b). While such analytical models are useful, they may be limited when different developmental trajectories are embedded in a data distribution. In the current study, being able to model different non-symbolic–symbolic magnitude ability change patterns in a single model, along with the associated cognitive factors/math abilities allowed us to represent a more comprehensive approach to modeling development (von Eye and Bergman, 2003).

We note, however, that our magnitude judgment tasks included stimuli from the so-called subitizing (*n* ≤ 4) and counting ranges that are thought to depend on different enumeration mechanisms (Reeve et al., 2012). It is possible that including items from the subitizing and counting ranges, either separately or in combination, may affect judgment responses. However, since similar response patterns occurred for comparison stimuli from the subitizing and counting ranges in both judgment tasks, we suggest the indices are arguably assessing a common underlying construct. Nonetheless, it is not always evident in magnitude judgment tasks whether performance reflects stimulus properties, task demands, or the construct under investigation (see Karolis et al., 2011 for a discussion).

## Conclusion

The current study replicated and extended Chew et al.’s (2016) findings. Indeed, the interpretive importance of replicating findings in latent class analysis research has recently been strongly emphasized (Hickendorff et al., 2018). The findings showed that identifiable differences in the profiles of relationships between non-symbolic and symbolic magnitude abilities could be extracted from a general distribution of these abilities. It also showed different change/stability pathways in these profiles over 1 year and that these were differentially associated with children’s math abilities. And, it also showed that VSWM and naming number abilities were differentially associated with the non-symbolic–symbolic magnitude ability profiles. While the present findings highlight the importance of paying attention to the developmental significance of different patterns of abilities over time which potentially represent typical and atypical developmental models, they should not be over-interpreted. The current study only examined stability/change in non-symbolic and symbolic magnitude abilities over 1 year. The issue of whether pathways converge later in time (i.e., reach equifinality), and the developmental math/cognitive implications of different pathways across extended time, is unable to be addressed in the present study and could usefully be the subject of future research.

## Author Contributions

CC collected and analyzed the data. RR, JF, and CC collaborated in writing the paper.

## Conflict of Interest Statement

The authors declare that the research was conducted in the absence of any commercial or financial relationships that could be construed as a potential conflict of interest.

## Supplementary Material

The Supplementary Material for this article can be found online at: https://www.frontiersin.org/articles/10.3389/fpsyg.2019.00441/full#supplementary-material

## References

[B1] Bailey D. H. Littlefield A. Geary D. C. (2012). The codevelopment of skill at and preference for use of retrieval-based processes for solving addition problems: individual and sex differences from first to sixth grades. *J. Exp. Child Psychol.* 113 78–92. 10.1016/j.jecp.2012.04.014 22704036 PMC3392429

[B2] Bakk Z. Tekle F. B. Vermunt J. K. (2013). Estimating the association between latent class membership and external variables using bias-adjustedthree-step approaches. *Sociol. Methodol.* 43 272–311. 10.1177/0081175012470644

[B3] Bartelet D. Vaessen A. Blomert L. Ansari D. (2014). What basic number processing measures in kindergarten explain unique variability in first-grade arithmetic proficiency? *J. Exp. Child Psychol.* 117 12–28. 10.1016/j.jecp.2013.08.010 24128690

[B4] Bergman L. R. Magnusson D. El-Khouri B. M. (2003). *Studying Individual Development in an Interindividual Context: A Person-Oriented Approach. Paths through Life*, 1st Edn, Vol. 4. London: Psychology Press. 10.1017/CBO9781107415324.004

[B5] Berteletti I. Lucangeli D. Piazza M. Dehaene S. Zorzi M. (2010). Numerical estimation in preschoolers. *Dev. Psychol.* 46 545–551. 10.1037/a0017887 20210512

[B6] Bouwmeester S. Verkoeijen P. P. J. L. (2012). Multiple representations in number line estimation: a developmental shift or classes of representations? *Cogn. Instr.* 30 246–260. 10.1080/07370008.2012.689384

[B7] Bugden S. Ansari D. (2011). Individual differences in children’s mathematical competence are related to the intentional but not automatic processing of Arabic numerals. *Cognition* 118 35–47. 10.1016/j.cognition.2010.09.005 20970782

[B8] Chen Q. Li J. (2014). Association between individual differences in non-symbolic number acuity and math performance: a meta-analysis. *Acta Psychol.* 148 163–172. 10.1016/j.actpsy.2014.01.016 24583622

[B9] Chew C. S. Forte J. D. Reeve R. A. (2016). Cognitive factors affecting children’s nonsymbolic and symbolic magnitude judgment abilities: a latent profile analysis. *J. Exp. Child Psychol.* 152 173–191. 10.1016/j.jecp.2016.07.001 27560661

[B10] Clark S. Muthen B. (2009). *Relating Latent Class Analysis Results to Variables not Included in the Analysis.* Available at: http://www.statmodel.com/download/relatinglca.pdf

[B11] Collins L. M. Lanza S. T. (2013). *Latent Class and Latent Transition Analysis: With Applications in the Social, Behavioral, and Health Sciences.* Hoboken, NJ: John Wiley & Sons.

[B12] Cotton S. M. Kiely P. M. Crewther D. P. Thomson B. Laycock R. Crewther S. G. (2005). A normative and reliability study for the Raven’s Coloured Progressive Matrices for primary school aged children from Victoria, Australia. *Pers. Individ. Dif.* 39 647–659. 10.1016/j.paid.2005.02.015

[B13] de Hevia M. D. (2016). *Core Mathematical Abilities in Infants: Number and Much More. Progress in Brain Research*, 1st Edn. Amsterdam: Elsevier B.V. 10.1016/bs.pbr.2016.04.014 27339008

[B14] de Hevia M. D. Vallar G. Girelli L. (2008). Visualizing numbers in the mind’s eye: the role of visuo-spatial processes in numerical abilities. *Neurosci. Biobehav. Rev.* 32 1361–1372. 10.1016/j.neubiorev.2008.05.015 18584868

[B15] De Smedt B. Gilmore C. K. (2011). Defective number module or impaired access? Numerical magnitude processing in first graders with mathematical difficulties. *J. Exp. Child Psychol.* 108 278–292. 10.1016/j.jecp.2010.09.003 20974477

[B16] De Smedt B. Noël M.-P. Gilmore C. Ansari D. (2013). How do symbolic and non-symbolic numerical magnitude processing skills relate to individual differences in children’s mathematical skills? A review of evidence from brain and behavior. *Trends Neurosci. Educ.* 2 48–55. 10.1016/j.tine.2013.06.001

[B17] De Smedt B. Verschaffel L. Ghesquière P. (2009). The predictive value of numerical magnitude comparison for individual differences in mathematics achievement. *J. Exp. Child Psychol.* 103 469–479. 10.1016/j.jecp.2009.01.010 19285682

[B18] Dehaene S. (1992). Varieties of numerical abilities. *Cognition* 44 1–42. 10.1016/0010-0277(92)90049-N 1511583

[B19] Dehaene S. (2007). “Symbols and quantities in parietal cortex: elements of a mathematical theory of number representation and manipulation,” in *Attention & Performance XXII. Sensori-Motor Foundation of Higher Cognition*, eds Haggard P. Rossetti Y. (Cambridge, MA: Harvard University Press), 527–574. 10.1093/acprof:oso/9780199231447.003.0024

[B20] Dehaene S. (2011). *The Number Sense: How the Mind Creates Mathematics, Revised and Updated Edition.* Oxford: Oxford University Press.

[B21] Dehaene S. Brannon E. M. (2011). *Space, Time and Number in the Brain: Searching for the Foundations of Mathematical Thought.* London: Academic Press.

[B22] Dehaene S. Cohen L. (1997). Cerebral pathways for calculation: double dissociation between rote verbal and quantitative knowledge of arithmetic. *Cortex* 33 219–250. 10.1016/S0010-9452(08)70002-9 9220256

[B23] Dehaene S. Izard V. Piazza M. (2005). *Control Over Non-Numerical Parameters in Numerosity Experiments.* Available at: Www.Unicog.Org

[B24] Dehaene S. Piazza M. Pinel P. Cohen L. (2003). Three parietal circuits for number processing. *Cogn. Neuropsychol.* 20 487–506. 10.1080/02643290244000239 20957581

[B25] Desoete A. Ceulemans A. De Weerdt F. Pieters S. (2012). Can we predict mathematical learning disabilities from symbolic and non-symbolic comparison tasks in kindergarten? Findings from a longitudinal study. *Br. J. Educ. Psychol.* 82 64–81. 10.1348/2044-8279.002002 21199482

[B26] Dowker A. (2008). Individual differences in numerical abilities in preschoolers. *Dev. Sci.* 11 650–654. 10.1111/j.1467-7687.2008.00713.x 18801119

[B27] Fazio L. K. Bailey D. H. Thompson C. A. Siegler R. S. (2014). Relations of different types of numerical magnitude representations to each other and to mathematics achievement. *J. Exp. Child Psychol.* 123C, 53–72. 10.1016/j.jecp.2014.01.013 24699178

[B28] Feigenson L. Dehaene S. Spelke E. (2004). Core systems of number. *Trends Cogn. Sci.* 8 307–314. 10.1016/j.tics.2004.05.002 15242690

[B29] Feigenson L. Libertus M. E. Halberda J. (2013). Links between the intuitive sense of number and formal mathematics ability. *Child Dev. Perspect.* 7 74–79. 10.1111/cdep.12019 24443651 PMC3891767

[B30] Friso-Van Den Bos I. Van Der Ven S. H. G. Kroesbergen E. H. Van Luit J. E. H. (2013). Working memory and mathematics in primary school children: a meta-analysis. *Educ. Res. Rev.* 10 29–44. 10.1016/j.edurev.2013.05.003

[B31] Geary D. C. (2000). From infancy to adulthood: the development of numerical abilities. *Eur. Child Adolesc. Psychiatry* 9(Suppl. 2), 11–16. 10.1007/s007870070004 11138899

[B32] Geary D. C. (2011). Cognitive predictors of achievement growth in mathematics: a 5-year longitudinal study. *Dev. Psychol.* 47 1539–1552. 10.1037/a0025510 21942667 PMC3210883

[B33] Geary D. C. Hoard M. K. (2005). “Learning disabilities in arithmetic and mathematics: theoretical and empirical perspectives,” in *Handbook of Mathematical Cognition* ed. Campbell J. I. D. (London: Psychology Press), 253–266.

[B34] Gebuis T. Cohen Kadosh R. De Haan E. Henik A. (2009). Automatic quantity processing in 5-year olds and adults. *Cogn. Process.* 10 133–142. 10.1007/s10339-008-0219-x 18607652

[B35] Gray S. A. Reeve R. A. (2014). Preschoolers’ dot enumeration abilities are markers of their arithmetic competence. *PLoS One* 9:e94428. 10.1371/journal.pone.0094428 24714052 PMC3979837

[B36] Gray S. A. Reeve R. A. (2016). Number-specific and general cognitive markers of preschoolers’ math ability profiles. *J. Exp. Child Psychol.* 147 1–21. 10.1016/j.jecp.2016.02.004 26985575

[B37] Halberda J. Mazzocco M. M. M. Feigenson L. (2008). Individual differences in non-verbal number acuity correlate with maths achievement. *Nature* 455 665–668. 10.1038/nature07246 18776888

[B38] Hickendorff M. Edelsbrunner P. A. McMullen J. Schneider M. Trezise K. (2018). Informative tools for characterizing individual differences in learning: latent class, latent profile, and latent transition analysis. *Learn. Individ. Dif.* 66 4–15. 10.1016/j.lindif.2017.11.001

[B39] Holloway I. D. Ansari D. (2009). Mapping numerical magnitudes onto symbols: the numerical distance effect and individual differences in children’s mathematics achievement. *J. Exp. Child Psychol.* 103 17–29. 10.1016/j.jecp.2008.04.001 18513738

[B40] Izard V. Sann C. Spelke E. S. Streri A. (2009). Newborn infants perceive abstract numbers. *Proc. Natl. Acad. Sci. U.S.A.* 106 10382–10385. 10.1073/pnas.0812142106 19520833 PMC2700913

[B41] Jordan N. C. Hansen N. Fuchs L. S. Siegler R. S. Gersten R. Micklos D. (2013). Developmental predictors of fraction concepts and procedures. *J. Exp. Child Psychol.* 116 45–58. 10.1016/j.jecp.2013.02.001 23506808

[B42] Karolis V. Iuculano T. Butterworth B. (2011). Mapping numerical magnitudes along the right lines: differentiating between scale and bias. *J. Exp. Psychol. Gen.* 140 693–706. 10.1037/a0024255 21767042

[B43] Kessels R. P. van Zandvoort M. J. Postma A. Kappelle L. J. de Haan E. H. (2000). The Corsi block-tapping task: standardization and normative data. *Appl. Neuropsychol.* 7 252–258. 10.1207/S15324826AN0704_8 11296689

[B44] Kolkman M. E. Kroesbergen E. H. Leseman P. P. M. (2013). Early numerical development and the role of non-symbolic and symbolic skills. *Learn. Instr.* 25 95–103. 10.1016/j.learninstruc.2012.12.001 30279672

[B45] Kyttälä M. Lehto J. E. (2008). Some factors underlying mathematical performance: the role of visuospatial working memory and non-verbal intelligence. *Eur. J. Psychol. Educ.* 23 77–94. 10.1007/BF03173141

[B46] Lanza S. T. Cooper B. R. (2016). Latent class analysis for developmental research. *Child Dev. Perspect.* 10 59–64. 10.1111/cdep.12163 PMC6914261 31844424

[B47] Laursen B. P. Hoff E. (2006). Person-centered and variable-centered approaches to longitudinal data. *Merrill Palmer Q.* 52 377–389. 10.1353/mpq.2006.0029

[B48] Libertus M. E. Feigenson L. Halberda J. (2013). Is approximate number precision a stable predictor of math ability? *Learn. Individ. Dif.* 25 126–133. 10.1016/j.lindif.2013.02.001 23814453 PMC3692560

[B49] Luwel K. Foustana A. Onghena P. Verschaffel L. (2013). The role of verbal and performance intelligence in children’s strategy selection and execution. *Learn. Individ. Dif.* 24 134–138. 10.1016/j.lindif.2013.01.010

[B50] Maloney E. A. Risko E. F. Preston F. Ansari D. Fugelsang J. (2010). Challenging the reliability and validity of cognitive measures: the case of the numerical distance effect. *Acta Psychol.* 134 154–161. 10.1016/j.actpsy.2010.01.006 20185118

[B51] Mammarella I. C. Lucangeli D. Cornoldi C. (2010). Spatial working memory and arithmetic deficits in children with nonverbal learning difficulties. *J. Learn. Disabil.* 43 455–468. 10.1177/0022219409355482 20375290

[B52] Mazzocco M. M. M. Feigenson L. Halberda J. (2011). Impaired acuity of the approximate number system underlies mathematical learning disability (dyscalculia). *Child Dev.* 82 1224–1237. 10.1111/j.1467-8624.2011.01608.x 21679173 PMC4411632

[B53] Moyer R. S. Landauer T. K. (1967). Time required for judgements of numerical inequality. *Nature* 215 1519–1520. 10.1038/2151519a0 6052760

[B54] Muthen B. Muthen L. K. (2000). Integrating person-centered and variable-centered analyses: growth mixture modeling with latent trajectory classes. *Alcohol. Clin. Exp. Res.* 24 882–891. 10.1111/j.1530-0277.2000.tb02070.x 10888079

[B55] OECD (2012). *Education at a Glance, Education at a Glance 2012: OECD Indicators.* Paris: OECD Publishing 10.1787/eag-2012-en

[B56] Paul J. M. Gray S. A. Butterworth B. L. Reeve R. A. (2018). Reading and math tests differentially predict number transcoding and number fact speed longitudinally: a random intercept cross-lagged panel approach. *J. Educ. Psychol.* 111 299–313. 10.1037/edu0000287

[B57] Paul J. M. Reeve R. A. (2016). Relationship between single digit addition strategies and working memory reflects general reasoning sophistication. *Learn. Instr.* 42 113–122. 10.1016/j.learninstruc.2016.01.011

[B58] Piazza M. (2010). Neurocognitive start-up tools for symbolic number representations. *Trends Cogn. Sci.* 14 542–551. 10.1016/j.tics.2010.09.008 21055996

[B59] Piazza M. Facoetti A. Trussardi A. N. Berteletti I. Conte S. Lucangeli D. (2010). Developmental trajectory of number acuity reveals a severe impairment in developmental dyscalculia. *Cognition* 116 33–41. 10.1016/j.cognition.2010.03.012 20381023

[B60] Piazza M. Izard V. (2009). How humans count: numerosity and the parietal cortex. *Neuroscientist* 15 261–273. 10.1177/1073858409333073 19436075

[B61] Price G. R. Palmer D. Battista C. Ansari D. (2012). Nonsymbolic numerical magnitude comparison: reliability and validity of different task variants and outcome measures, and their relationship to arithmetic achievement in adults. *Acta Psychol.* 140 50–57. 10.1016/j.actpsy.2012.02.008 22445770

[B62] Ratcliff R. Thompson C. A. Mckoon G. (2015). Modeling individual differences in response time and accuracy in numeracy. *Cognition* 137 115–136. 10.1016/j.cognition.2014.12.004 25637690 PMC4353499

[B63] Raven J. Court J. H. Raven J. C. (1995). *Coloured Progressive Matrices.* Oxford: Oxford Psychologists Press.

[B64] Reeve R. Gray S. Butterworth B. Paul J. (2018). Variability in single digit addition problem-solving speed over time identifies typical, delay and deficit math pathways. *Front. Psychol.* 9:1498. 10.3389/FPSYG.2018.01498 30154754 PMC6102488

[B65] Reeve R. Reynolds F. Humberstone J. Butterworth B. (2012). Stability and change in markers of core numerical competencies. *J. Exp. Psychol. Gen.* 141 649–666. 10.1037/a0027520 22409662

[B66] Rousselle L. Noël M.-P. (2007). Basic numerical skills in children with mathematics learning disabilities: a comparison of symbolic vs non-symbolic number magnitude processing. *Cognition* 102 361–395. 10.1016/j.cognition.2006.01.005 16488405

[B67] Sasanguie D. De Smedt B. Defever E. Reynvoet B. (2012a). Association between basic numerical abilities and mathematics achievement. *Br. J. Dev. Psychol.* 30 344–357. 10.1111/j.2044-835X.2011.02048.x 22550952

[B68] Sasanguie D. Van den Bussche E. Reynvoet B. (2012b). Predictors for mathematics achievement? Evidence from a longitudinal study. *Mind Brain Educ.* 6 119–128. 10.1111/j.1751-228X.2012.01147.x

[B69] Sasanguie D. Defever E. Maertens B. Reynvoet B. (2014). The approximate number system is not predictive for symbolic number processing in kindergarteners. *Q. J. Exp. Psychol.* 67 271–280. 10.1080/17470218.2013.803581 23767979

[B70] Sasanguie D. Göbel S. M. Moll K. Smets K. Reynvoet B. (2013). Approximate number sense, symbolic number processing, or number–space mappings: what underlies mathematics achievement? *J. Exp. Child Psychol.* 114 418–431. 10.1016/J.JECP.2012.10.012 23270796

[B71] Schneider M. Beeres K. Coban L. Merz S. Susan Schmidt S. Stricker J. (2016). Associations of non-symbolic and symbolic numerical magnitude processing with mathematical competence: a meta-analysis. *Dev. Sci.* 20:e12372. 10.1111/desc.12372 26768176

[B72] Siegler R. S. (1987). The perils of averaging data over strategies: an example from children’s addition. *J. Exp. Psychol. Gen.* 116 250–264. 10.1037/0096-3445.116.3.250

[B73] Siegler R. S. (2016). Continuity and change in the field of cognitive development and in the perspectives of one cognitive developmentalist. *Child Dev. Perspect.* 10 128–133. 10.1111/cdep.12173

[B74] Träff U. (2013). The contribution of general cognitive abilities and number abilities to different aspects of mathematics in children. *J. Exp. Child Psychol.* 116 139–156. 10.1016/j.jecp.2013.04.007 23773916

[B75] Trezise K. Reeve R. A. (2014). Cognition-emotion interactions: patterns of change and implications for math problem solving. *Front. Psychol.* 5:840. 10.3389/fpsyg.2014.00840 25132830 PMC4116786

[B76] Vanbinst K. Ceulemans E. Ghesquière P. De Smedt B. (2015a). Profiles of children’s arithmetic fact development: a model-based clustering approach. *J. Exp. Child Psychol.* 133 29–46. 10.1016/j.jecp.2015.01.003 25731679

[B77] Vanbinst K. Ghesquière P. De Smedt B. (2015b). Does numerical processing uniquely predict first graders’ future development of single-digit arithmetic? *Learn. Individ. Differ.* 37 153–160. 10.1016/j.lindif.2014.12.004

[B78] Vermunt J. K. Magidson J. (2013a). *Latent Gold 5.0 Upgrade Manual 1.* Belmont, MA: Statistical Innovations Inc.

[B79] Vermunt J. K. Magidson J. (2013b). *Technical Guide for Latent GOLD 5.0: Basic, Advanced, and Syntax.* Belmont, MA: Statistical Innovations Inc.

[B80] Vermunt J. K. Magidson J. (2015). *Upgrade Manual for Latent GOLD 5.1.* Belmont, MA: Statistical Innovations.

[B81] von Eye A. Bergman L. R. (2003). Research strategies in developmental psychopathology: dimensional identity and the person-oriented approach. *Dev. Psychopathol.* 15 553–580. 10.1017/S0954579403000294 14582932

[B82] Xenidou-Dervou I. Molenaar D. Ansari D. van der Schoot M. van Lieshout E. C. D. M. (2016). Nonsymbolic and symbolic magnitude comparison skills as longitudinal predictors of mathematical achievement. *Learn. Instr.* 50 1–13. 10.1016/j.learninstruc.2016.11.001

[B83] Zorzi M. Priftis K. Umiltà C. (2002). Brain damage: neglect disrupts the mental number line. *Nature* 417 138–139. 10.1038/417138a 12000950

