# Supplementary material for: Implications of Change/Stability Patterns in Children’s Non-symbolic and Symbolic Magnitude Judgment Abilities Over One Year: A Latent Transition Analysis
Source: Front Psychol. 2019 Mar 5;10:441. doi: 10.3389/fpsyg.2019.00441 (PMC6411817; doi:10.3389/fpsyg.2019.00441)
Supplement: Supplementary file 1 [file Data_Sheet_1.docx]

Supplementary Material for *Change/stability patterns in children’s nonsymbolic and symbolic magnitude judgment abilities: A latent transition analysis*

**S1. Results**

**Nonsymbolic/symbolic magnitude judgment accuracy and RT as a function of ratios and grades at Time 1 and 2**

Table S1

*Nonsymbolic Response Time and Accuracy as a Function of Ratio and Grade at Time 1 and 2*

|  | Grade at Time 1 | | | | | | | | | |
| --- | --- | --- | --- | --- | --- | --- | --- | --- | --- | --- |
|  | Kindergarten | | Year 1 | |  | Kindergarten | | | Year 1 | |
|  | Response time (ms) | | | |  | Accuracy | | | | |
| Ratios | *M* | *SD* | *M* | *SD* |  | *M* | *SD* | *M* | | *SD* |
| .10-.19 | 1237.04 | 392.64 | 1088.58 | 309.84 |  | .90 | .16 | .97 | | .06 |
| .20-.29 | 1279.15 | 431.25 | 1077.41 | 291.30 |  | .89 | .15 | .96 | | .06 |
| .30-.39 | 1394.48 | 405.87 | 1146.56 | 374.28 |  | .84 | .21 | .94 | | .09 |
| .40-.49 | 1464.05 | 430.95 | 1278.61 | 381.27 |  | .88 | .17 | .94 | | .11 |
| .50-.59 | 1523.89 | 476.70 | 1367.38 | 314.63 |  | .84 | .17 | .92 | | .10 |
| .60-.69 | 1487.49 | 492.46 | 1408.38 | 378.16 |  | .77 | .19 | .88 | | .13 |
| .70-.79 | 1742.55 | 690.68 | 1642.96 | 523.05 |  | .70 | .21 | .79 | | .20 |
| .80-.89 | 1729.04 | 744.12 | 1854.83 | 747.23 |  | .55 | .21 | .55 | | .23 |
|  | Grade at Time 2 | | | | | | | | | |
|  | Year 1 | | Year 2 | |  | Year 1 | | Year 2 | | |
| Ratios | *M* | *SD* | *M* | *SD* |  | *M* | *SD* | *M* | | *SD* |
| .10-.19 | 953.45 | 214.38 | 818.23 | 211.16 |  | .97 | .09 | .97 | | .07 |
| .20-.29 | 994.00 | 202.95 | 820.35 | 287.72 |  | .95 | .09 | .98 | | .05 |
| .30-.39 | 997.19 | 215.43 | 914.73 | 258.92 |  | .96 | .08 | .98 | | .05 |
| .40-.49 | 1004.33 | 274.03 | 929.37 | 306.55 |  | .97 | .07 | .97 | | .07 |
| .50-.59 | 1160.09 | 331.36 | 986.30 | 334.32 |  | .94 | .08 | .98 | | .04 |
| .60-.69 | 1176.88 | 290.89 | 1037.65 | 333.94 |  | .93 | .10 | .94 | | .08 |
| .70-.79 | 1431.37 | 401.83 | 1204.30 | 431.20 |  | .90 | .12 | .90 | | .10 |
| .80-.89 | 1542.23 | 523.90 | 1397.02 | 692.28 |  | .72 | .19 | .75 | | .18 |

Table S2

*Symbolic Response Time and Accuracy as a Function of Ratio and Grade at Time 1 and 2*

|  | Grade at Time 1 | | | | | | | | | |
| --- | --- | --- | --- | --- | --- | --- | --- | --- | --- | --- |
|  | Kindergarten | | Year 1 | |  | Kindergarten | | | Year 1 | |
|  | Response time (ms) | | | |  | Accuracy | | | | |
| Ratios | *M* | *SD* | *M* | *SD* |  | *M* | *SD* | *M* | | *SD* |
| .10-.19 | 1132.25 | 460.57 | 1060.96 | 214.65 |  | .90 | .18 | .94 | | .09 |
| .20-.29 | 1235.57 | 491.37 | 1051.73 | 283.04 |  | .86 | .19 | .93 | | .11 |
| .30-.39 | 1204.61 | 453.08 | 1120.89 | 306.48 |  | .87 | .19 | .92 | | .12 |
| .40-.49 | 1385.74 | 459.43 | 1120.71 | 367.10 |  | .84 | .20 | .91 | | .12 |
| .50-.59 | 1413.66 | 499.94 | 1232.22 | 352.97 |  | .82 | .19 | .88 | | .11 |
| .60-.69 | 1473.73 | 554.63 | 1349.26 | 373.37 |  | .77 | .19 | .84 | | .15 |
| .70-.79 | 1529.51 | 506.11 | 1333.10 | 477.09 |  | .75 | .21 | .83 | | .14 |
| .80-.89 | 1522.28 | 559.41 | 1321.96 | 381.32 |  | .72 | .19 | .81 | | .13 |
|  | Grade at Time 2 | | | | | | | | | |
|  | Year 1 | | Year 2 | |  | Year 1 | | Year 2 | | |
| Ratios | *M* | *SD* | *M* | *SD* |  | *M* | *SD* | *M* | | *SD* |
| .10-.19 | 945.02 | 248.26 | 827.41 | 181.52 |  | .97 | .06 | .97 | | .05 |
| .20-.29 | 1051.70 | 213.61 | 858.31 | 286.22 |  | .96 | .07 | .96 | | .06 |
| .30-.39 | 989.93 | 242.24 | 850.19 | 244.63 |  | .95 | .09 | .97 | | .07 |
| .40-.49 | 1150.93 | 373.34 | 915.51 | 266.96 |  | .91 | .12 | .95 | | .10 |
| .50-.59 | 1166.49 | 242.18 | 997.02 | 286.46 |  | .92 | .09 | .92 | | .10 |
| .60-.69 | 1255.40 | 353.41 | 1069.05 | 306.31 |  | .89 | .10 | .90 | | .11 |
| .70-.79 | 1227.66 | 237.59 | 1039.02 | 291.80 |  | .85 | .15 | .91 | | .10 |
| .80-.89 | 1197.10 | 341.65 | 1043.72 | 349.75 |  | .85 | .12 | .84 | | .10 |

One-way ANOVAs and Bonferroni-corrected post hoc comparisons showed profiles differed from each other in nonsymbolic and symbolic magnitude, accuracy and RT. For nonsymbolic magnitude accuracy, *Welch*’s *F* (3, 65) = 43.87, *p*<.001, *η²* = .43 –*Welch’s* F is reported because of a violation in the homogeneity of variance assumption; and for symbolic magnitude accuracy, *Welch*’s *F* (3, 66) = 24.74, *p*<.001, *η²* = .44. Profile 4 (nonsymbolic: *M*=.71, *SD*=.17; symbolic: *M*=.66, *SD*=.16) was less accurate than all other profiles on both nonsymbolic and symbolic magnitude (all *p*s<.001). Profile 2 (nonsymbolic: *M*=.95, *SD*=.03; symbolic: *M*=.93, *SD*=.05) was more accurate than all other profiles on both nonsymbolic and symbolic magnitude (all *p*s<.001; except Profile 1 on symbolic magnitude accuracy). Profile 3 (nonsymbolic: *M*=.84, *SD*=.09; symbolic: *M*=.88, *SD*=.07) was less accurate than Profiles 1 (nonsymbolic: *M*=.90, *SD*=.05; symbolic: *M*=.92, *SD*=.04) and 2 but more accurate than Profile 4 both nonsymbolic and symbolic magnitude (all *p*s<.001).

For nonsymbolic magnitude RT, *Welch*’s *F* (3, 66) = 87.52, *p*<.001, *η²* = .47; and for symbolic magnitude RT, *Welch*’s *F* (3, 66) = 117, *p*<.001, *η²* = .47. Profiles 1 (nonsymbolic: *M*=1209.1, *SD*=169.19; symbolic: *M*=1168.06, *SD*=137.08) and 2 (nonsymbolic: *M*=913.27, *SD*=130.47; symbolic: *M*=870.6, *SD*=127.27) were faster than Profiles 3 (nonsymbolic: *M*=1517.05, *SD*=317.52; symbolic: *M*=1444.25, *SD*=221.05) and 4 (nonsymbolic: *M*=1542.48, *SD*=529.67; symbolic: *M*=1426.15, *SD*=622.28) both nonsymbolic and symbolic magnitude RT (all *p*s<.001). Profile 2 was also faster than Profile 3 on both nonsymbolic and symbolic magnitude RT (all *p*s<.001).

Table S3

*Mean Accuracy and Median RT for NSM-SM Profiles at Time 1 and Time 2*

|  | NSM Accuracy | SM Accuracy | NSM RT | SM RT |
| --- | --- | --- | --- | --- |
|  | Time 1 | | | |
| Profile 1 | .89 | .92 | 1263.64 | 1135.72 |
| Profile 2 | .93 | .85 | 859.91 | 917.42 |
| Profile 3 | .84 | .88 | 1540.15 | 1471.63 |
| Profile 4 | .71 | .66 | 1636.15 | 1292.33 |
| Overall Mean | .84 | .85 | 1331.45 | 1275.53 |
|  | Time 2 | | | |
| Profile 1 | .92 | .92 | 1157.44 | 1210.64 |
| Profile 2 | .95 | .93 | 895.61 | 859.93 |
| Profile 3 | .85 | .85 | 1450.63 | 1430.80 |
| Profile 4 | - | - | - | - |
| Overall Median | .93 | .92 | 1011.79 | 995.18 |

*Note*. NSM, nonsymbolic magnitude; SM, symbolic magnitude.
